# In Vivo Screening and Antidiabetic Potential of Polyphenol Extracts from Guava Pulp, Seeds and Leaves

**DOI:** 10.3390/ani10091714

**Published:** 2020-09-22

**Authors:** Hassan Shabbir, Tusneem Kausar, Sobia Noreen, Hafeez ur Rehman, Ashiq Hussain, Qingrong Huang, Adil Gani, Shiwei Su, Asad Nawaz

**Affiliations:** 1 Institute of Food Science and Nutrition, Sargodha University, Sargodha 40100, Pakistan; thehassanmirza@gmail.com (H.S.); Sobia.noreen@uos.edu.pk (S.N.); Hafeez1386@gmail.com (H.u.R.); ashiqft@gmail.com (A.H.); 2 Department of Food Science, Rutgers State University, 65 Dudley Road, New Brunswick, NJ 08901, USA; qhuang@aesop.rutgers.edu (Q.H.); adil.gani@gmail.com (A.G.); Shiwei_su@yahoo.com (S.S.); 3 Jiangsu Key Laboratory of Crop Genetics and Physiology, Key Laboratory of Plant Functional Genomics of the Ministry of Education, College of Agriculture, Yangzhou University, Yangzhou 225009, China; 007298@yzu.edu.cn

**Keywords:** guava, polyphenol, diabetes, blood serum, biochemical parameters

## Abstract

**Simple Summary:**

The incidence of diabetes has risen from 151 million people in 2000 to 463 million in 2019, with 4.2 million estimated deaths in 2019, and over 700 million people will be affected with diabetes worldwide by 2045. Thus, the screening of anti-diabetic plants is inevitable in order to overcome diabetes. This study has investigated the anti-diabetic potential of polyphenol extracts from guava leaves, seeds and pulp using an albino rat model. The in vivo study has reported that polyphenols from leaves and pulp have an ability to improve diabetic parameters, such as insulin, blood glucose and triglycerides levels. The improvement in blood parameters is also an indication of these extracts that are valuable for diabetes management. The findings of this study reveal that polyphenols from guava leaves, pulp and seeds can be used for drug development.

**Abstract:**

The present study investigates the antidiabetic potential of polyphenol extracts purified from guava pulp, seeds and leaves using an in vivo experiment on albino rats. The polyphenols from guava pulp, seeds and leaves were extracted using methanol solvent and the sonication method while being evaluated by total phenolic contents and radical scavenging activity assay. The proximate composition of powders revealed that ash, protein and total sugars were significantly (*p* < 0.05) higher in leaves and seeds, while vitamin C was highest in pulp. Total phenolic and antioxidant activities were highest in pulp followed by leaves and seeds. The findings of feed intake and body gain revealed that the supplementation of polyphenols, especially from pulp, significantly (*p* < 0.05) increased the feed intake, which resulted in increased body weight. Moreover, total cholesterol (TC) and low-density lipoprotein (LDL) levels were significantly (*p* < 0.05) decreased, while the level of high-density lipoprotein (HDL) was increased in groups fed with polyphenols from guava pulp compared to both (+ive and –ive) control groups. Furthermore, blood glucose and triglycerides were significantly (*p* < 0.05) decreased in supplemented groups compared to the control group of diabetes mice, which resulted in the inhibition of α-amylase and glucose transport. Besides this, packed cell volume (PCV), mean corpuscular volume (MCV), hemoglobin, red blood cells (RBCs), white blood cells (WBCs) and platelet levels were increased significantly (*p* < 0.05) in pulp’s extract followed by leaves and seeds compared to both control groups. Overall, the antidiabetic potential of different extracts was in the following order: pulp > leaves > seeds. The findings suggest the feasibility of adding 200–250 mg/kg.bw of polyphenol extracts of pulp as an alternative to diabetic drugs.

## 1. Introduction

Diabetes has become an increasingly persistent global concern, with the incidence growing rapidly due to changes in modern lifestyles, including diet and environmental changes. It is estimated that the incidence of diabetes has risen from 108 million people in 1980 to 425 million in 2017, with 1.6 million estimated deaths being recorded in 2016 [1]. The prevalence of diabetes has been rising rapidly in adults worldwide in both developed and developing countries. It has been projected that over 600 million people will be affected with diabetes by 2040 [1]. The incidence of diabetes has also been observed in animals such as dogs and cats [1]. Diabetes in animals is more severe than human as more than 50% of cats and dogs are obese that is the leading cause of diabetes. Almost half of the deaths resulting from diabetes are attributed to high levels of blood glucose. Thus, the prevention, treatment and mitigation of diabetes have attracted considerable research resources worldwide. However, despite these efforts, problems associated with type 2 diabetes are becoming worse due to lifestyle changes [2]. Although, various drugs have been discovered in order to control diabetes, however, some have side effects [3]; thus, screenings of antidiabetic agents, especially the functional foods, are inevitable.

Guava (*Psidium guajava* L.), known as a tropical fruit, has numerous commercial applications owing to its taste, flavor and aroma. It contains a broad spectrum of phytochemicals, including polysaccharides, alkaloids, glycosides, vitamins (it contains four times more vitamin C than citrus), essential oils, minerals, enzymes, proteins [4], flavonoids [5], tannins, and saponins [6]. Various pharmacological uses have been reported from its parts, such as the antiseptic effect of leaves, fresh fruit and tea from leaves in order to treat diarrhea, dysentery, diabetes mellitus and others [4,5,7]. The leaves (having phenolic compounds) and bark of the *P. guajava* tree have an extensive history of medicinal uses that are still active today [6].

The polyphenols and flavonoids from fruits and vegetables have attracted considerable attention due to the wide range of therapeutic activities [8]. Bioactive compounds from plant sources, especially polyphenols have been proposed to affect the glucose metabolism. Polyphenols play an important role in the control of diabetes by inhibiting the absorption of glucose from small intestine, enhancing the secretion of insulin and the controlled release of glucose from liver [6]. Among these, flavonoids are responsible for their therapeutic activities; much of a guava’s therapeutic activity and antibacterial activity have been attributed to these flavonoid compounds [5]. Guava also has antioxidant characteristics due to the polyphenols found in leaves, peel and immature fruits. However, different varieties (red or white flesh) of guava have different nutritional components. Guava contains carotenoids and polyphenols, two main antioxidant pigments, which have a high antioxidant value in plant food [9]. Since these pigments can produce the color of peel and pulp, red orange guava contains more pigments than yellow green guava, such as, polyphenols, carotenoids, and vitamin A (a retinoid source) [10]. Polyphenols and antioxidants bind the α-amylase and α-glucosidase, thus acting as a functional food [11,12].

The importance of analytical methods regarding the measurement of feedstuffs is of high interest and plays a vital role for its bioavailability. Taking into account the case of polyphenols (having pronounced complexity) regarding their quantification and bioavailability, multidisciplinary approaches are necessary [5]. Analytical methods to assess feedstuffs, such as in vitro, in vivo, and clinical trials, are the widely used and the most acceptable methods around the globe. In addition, the quantification, extraction and purification techniques for a highly bioavailable feedstuff are crucial factors that evaluate the efficiency of feedstuffs. Among these, in vitro clinical trials, especially rat models, for diabetes are considered as reliable techniques. Thus, the exploration of economical and suitable methods of extraction and their bioavailability is the main focus of scientists. 

Keeping in view the importance of guava, the screening of its different parts, such as pulp, seeds and leaves, for antioxidant and antidiabetic potential is necessary. Thus, the present study was aimed at the extraction of polyphenols from three parts of guava—the fruit pulp, seeds and leaves. The evaluations of total phenolic compounds and antioxidant properties were assessed using 2,2-diphenyl-1-picrylhydrazyl (DPPH) free radical scavenging assay. Two doses (200, 250 mL/kg/bw) of polyphenol extracts were selected for three weeks. The feedstuffs (phenolic extracts) were assessed for anitdiabetic potential using various diabetic parameters, such as blood glucose, blood insulin, total cholesterol (TC), white blood cells (WBCs), red blood cells (RBCs) and platelets. The findings of this study will help to find the suitability of guava as an antidiabetic agent and an optimum ratio necessary for a proper drug development. 

## 2. Materials and Methods 

### 2.1. Plant Samples

Guava (*Psidiumguajava)* fruits and leaves were collected from University College of Agriculture, University of Sargodha (UOS), Sargodha, Punjab, Pakistan, and transported to the Institute of Food Science and Nutrition, UOS. These were thoroughly washed under tap water in order to remove dirt, dust, microbes and pesticide residue on the surface. The peeling of fruits and the separation of seeds from pulp were done by stainless steel knife followed by drying in the hot air oven at 40 °C. After drying, these were ground to a fine powder by passing through a mesh of sieve size 0.5 mm (mesh 35). Guava leaves, seeds and pulp powders were packed in airtight polyethylene zip bags and stored at ambient temperature for further use. 

### 2.2. Proximate Composition

The moisture, fat, ash, fiber and protein contents of all powders were determined following the standard method of AOAC (2000) [13] using oven drying, soxhlet apparatus, muffle furnace, digestion and Kjeldhal methods (nitrogen factor, 5.64), respectively. 

### 2.3. Extraction of Polyphenols

The extraction of polyphenols from guava leaves, seeds and pulp was done by maceration and ultrasound-assisted extraction methods following the protocol of Both, Chemat [14], with slight modifications. Polyphenols from guava leaves, seeds and pulp were extracted individually using methanol as a solvent, a concentration of 80%, a solvent ratio of 1:20 and an extraction temperature of 30 °C. Briefly, 5 g of each powder sample was extracted using sonicator (Transsonic 700 Elma) at 35 kHz frequency for 50 min by specific solvent in shaking water bath (Tecator 1024) for 24 h. After this, filtration of extract was done using Whatman filter paper 1 followed by centrifugation at 5000 rpm for 10 min. After centrifugation, the supernatant was collected and the solvent was evaporated using rotary evaporator (RE501, Gongyi Yuhua Instrument Co., Ltd., Gongyi, Henan, China) using vacuum at 45 °C to obtain dry extract, which was collected in glass bottles and stored at refrigerated temperature (4 °C). The percent yield of each extract was determined by dividing the weight of extract over sample weight and multiplied by 100. 

### 2.4. Total Polyphenol and DPPH Radical Scavenging Activity Assay

Total phenolic content of guava leaves, seeds and pulp extracts were measured by the Folin–Ciocalteau method elaborated by Qureshi, Stecher [15], with slight modifications. Briefly, aliquots (4 mL) of methanol extract was diluted in water and added into Folin-Ciocalteau reagent (0.25 mL) with the addition of 0.75 mL sodium carbonate (20%). This solution was magnetically stirred and placed in a dark place for 2 h. The absorbance of solution was recorded at 765 nm with UV-VIS Spectrophotometer (Agilent 8453). The absorbance was measured together with control consisted of water and reagent. The stock solution was also prepared simultaneously to make a standard curve. Total polyphenol content was expressed as mg of gallic acid equivalent (GAE)/g of extract. The antioxidant activity of polyphenol extract was determined using DPPH (1,1-diphenyl-2-picryl-hydrazyl) assay according to the method of Safdar et al. [16] with minor modifications. Concisely, 3 mL of methonolic extract was mixed with 100 µL of sample at various concentrations (25–400 µL/mL), stirred and placed at a dark place for 20 min. The absorbance was noted at 517 nm. A blank (parallel control without extract) sample and standard ascorbic acid were also analyzed in a similar way. The formula for the calculation of scavenging activity was as followed:

%inhibition of DPPH radical = Ac − As/Ac × 100(1)

where Ac indicates the absorbance of control, while As represents the absorbance of sample.

### 2.5. Experimental Design for Biological Studies

Albino rats of either sex weighing between 190 ± 10 g were used for biological studies to be done at animal house, Department of Pharmacy, UOS, Sargodha, Pakistan. Albino rats were made diabetic using a subcutaneous injection of 200 mg Alloxan/kg/bw by means of 0.1 M citrate buffer, pH 4.5. The rats were classified as diabetic based on higher blood glucose level (16 mM, continuously monitored for 4 days after the supplementation of alloxan). Animals were randomly divided into 8 groups having 5 animals in each group. For an initial period of one week, normal diet was given to the rats to adjust them in the environment. Two concentrations (200 and 250 mg/kg/bw) of all extracts were administrated along with experimental diet (same for all groups). The composition of experimental/standard diet was: Casein 20% (*w*/*w* of total diet), L-cystine 0.4%, corn starch 40%, soluble starch 12%, sucrose 10%, cellulose 5%, mixed vitamins 1%, choline bitartrate 0.25%, mixed minerals 4% and corn oil 7%. The extracts were mixed with basil diet and supplemented to rats on daily basis. Group 1: healthy mice fed on basal diet without intervention of diabetes was served as −ive control. Group 2: Diabetic mice fed on basal diet served as +ive control. Groups 3 and 4 were fed a basil diet with the supplementation of pulp extract at a rate of 200 and 250 mL/kg.bw, respectively. Groups 5, and 6 were fed with a basal diet along with 200, and 250 mL/kg/bw of leave extract while Groups 7 and 8 were fed with seed extract: 200, 250 mL/kg/bw, respectively. Groups 2 to 8 all were diabetic mice. During the experimental period (21 days), the temperature and relative humidity of the animal room were 23 ± 2 °C and 55 ± 5%, respectively, and the light–dark period was 12:12 h. During 3 weeks of the trial, the normal group and the experimental groups were given same diets, respectively, to evaluate the effect of individualized treatment on the selected parameters, such as, serum profile, glucose and insulin levels. Physiological parameters such as mental status, consciousness, food intake and remaining food were monitored on a daily basis. Food intake was recorded by subtracting the remaining (food that was not consumed) from total food at the end of each day. During the feeding period (3 weeks), no mortality was found in all treatment groups indicating the safe use and non-cytotoxicity of polyphenolic extracts. The rats’ blood samples were collected at 0, 7, 14, and 21 days for analysis.

### 2.6. Physiological Parameters

Feed and drink intake was recorded on daily basis during the entire experimental period, while body weight was measured at the end of each week following the protocol of the previous study [17]. Other physiological parameters such as mental status, food intake and consciousness were monitored regularly. At the termination of the animal study, blood samples of rats were collected through cardiac puncture in ethylene diaminetetra acetic acid (EDTA)-coated tubes for hematological study and non-coated tubes to measure serum profile, glucose and insulin levels using Glucometer.

### 2.7. Blood Serum and Biochemical Analysis

The rats were killed using a rabbit stand of slaughtering and blood was withdrawn from a jugular vein with the help of syringe and placed in different blood collection tubes of 5 mL containing EDTA to separate serum. Serum was sent to diagnostic laboratory for the estimation of serum creatinine, glucose and urea at the start of study and after every week following the previously described method [18]. The values are displayed in the form of mean and standard deviation. Centrifugation of blood was done in order to separate the serum at 4000 rpm for 10 min, which was used further for biochemical studies. Triglycerides, TC, high-density lipoprotein (HDL), low-density lipoprotein (LDL), packed cell volume (PCV), and mean corpuscular volume (MCV), hemoglobin level, RBC, WBC and platelets were measured while the mean and standard deviations were reported. Briefly, in order to analyze triglycerides and cholesterol, 2.5 µL of blood serum was added to 250 µL of reagent, and incubation (at 37 °C) was carried out for 10 min. After this, absorbance was measured at 150 nm using analyzer (7600-020, Clinical analyzer, Hitachi High-Technologies, Tokyo, Japan) following the standard protocol of manufacturers against blank and standard. In order to calculate the LDL and HDL of blood serum, 2.5 µL of standard or serum was added to 180 µL of reagent A followed by incubation (at 37 °C) for 5 min, while the absorbance was recorded at 546 nm using a blank sample. Later on, reagent B was added to each sample and incubated further for 5 min, while absorbance was measured at 546 nm using blank sample. 

Blood insulin was determined using an ELISA kit following the standard manufacturer’s instructions as follows: 40 µL of serum was shaken with 10 µL of insulin antibody and 50 µL of streptavidin horseradish peroxidase on a plate that was covered with sealed membrane followed by incubation for 60 min. Later on, the membrane was taken off; the subsequent liquid was poured out. Chromogen solution (50 µL, solution A) and chromogen solution (50 µL, solution B) were added into it and incubated further for 10 min in a dark place. At the end, 50 µL of stop solution was mixed with it in order to stop the reaction. The absorbance of the solution was recorded using EnSpire^TM^ Multimode Plate Reader (PerkinElmer, Waltham, MA, USA) at 450 nm using a blank. For a linear regression equation, the standard of insulin at various concentrations was prepared. 

For RBC, WBC, platelets and haemoglobin levels in blood, an automated manner analyzer automated manner (Abacus Junior Vet, Diatron MI Zrt., Budapest, Hungary) was used to measure respective values. All values were calculated in percentage and expressed as mean and standard deviation of triplicates. 

### 2.8. Statistical Analysis

All analyses were done in triplicates. One-way analysis of variance (ANOVA) and a Duncan Multiple Range’s test (DMRT) were conducted at a significant level of *p* < 0.05. All graphs were plotted using Origin Pro. 16. 

## 3. Results

### 3.1. Proximate Analysis 

The results of proximate composition are displayed in Table 1. Moisture contents were highest for guava leaf powder followed by pulp and seeds. Ash content was highest in leaf powder followed by seeds and pulp. Crude fat was highest in the following order: seeds > pulp > leaves, while protein content was in the following order: leaves > seeds > pulp. Meanwhile, fiber content was highest in leaves, while it was lowest in seeds. Carbohydrate content was highest in the leaves followed by pulp and seeds. The results of vitamin C (Table 1) show that pulp had the highest content, while seeds had the lowest level. 

### 3.2. Polyphenols of Guava Parts

The results of total phenolic compounds are shown in Table 1. The results depict that maximum total polyphenol contents were noticed for guava leaves followed by pulp and seeds. The total antioxidant activity that represents the ability of the plants to inhibit the bleaching effect of ascorbic acid measured and compared with the control possessing no antioxidant component. The findings reveal that the maximum antioxidant capacity was found in following order: pulp > leaves > seeds. 

The DPPH radical scavenging activity test is a widely used technique around the globe to assess the antioxidant potential of different plant extracts. According to the results (Table 1), the scavenging activity of pulp was the highest, which was attributed to the composition of pulp as vitamin C was also highest in pulp. This might be due to the extraction method especially the methanol extraction method that has widely been used around the world due to the high extraction efficiency and high antioxidant value. Thus, extracts from pulp followed by leaves and seeds were found to be having more antioxidant potential, which will ultimately affect the diabetic parameters. 

### 3.3. Feed intake and Body Weight

**Figure 1:**
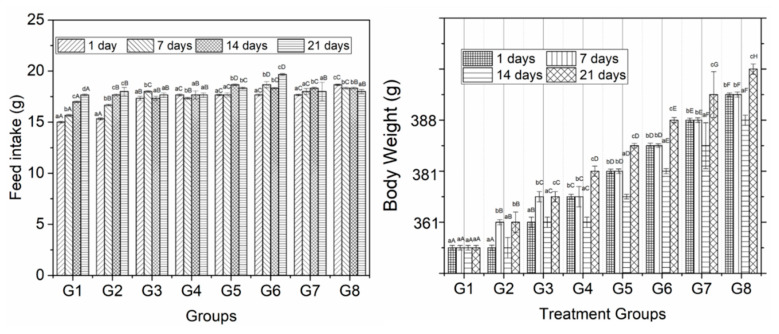
Feed intake and body weight of mice supplemented with different concentrations of guava leaves, seeds and pulp extract. Feed intake was calculated by subtracting leftovers from total food fed on each day. Error bars show the standard deviation (SD) of three replicate measurements. Uppercase letters (A–C) on error bars show significant differences (*p* < 0.05) in treatment times within the same treatments. Lowercase letters (a–c) show the significant difference (*p* < 0.05) among treatments within the same time.

The phenolic extracts of guava leaves, pulp and seeds were supplemented to diabetic mice and compared bio-chemical parameters with a positive and negative control for 3 weeks. The results of feed intake are shown in Figure 1. The findings reveal a significant change in the feed intake of experimental rats among different diet groups during the entire study interval. The results were also found to be significant at 0 and 21 days of a feeding interval. Mean values for the feed intake of group rats showed a significant effect. Group 6 showed the highest value (18.33 g). Group 1 at 21 days of trial interval showed the lowest values (16.33 g) of feed intake. 

The results of body weight are presented in Figure 1 that showed significant results in groups and study duration from 0 to 21 days. The maximum values (396.25 g) were observed in group 8. The minimum values (305 g) were observed in group 1 and group 2. However, regarding comparison within an experimental period, weight gain increments were high at 7 and 21 days. The gain in weight was more in 2 and 4 weeks, whereas, from week 1 to 3, the weight gain was less for both control and experimental diets. Among the difference between the weekly weights, the control diet albino rats gained more weight when compared with experimental diet albino rats except in weeks 2 and 3. 

### 3.4. Serum Lipid Profile

**Figure 2:**
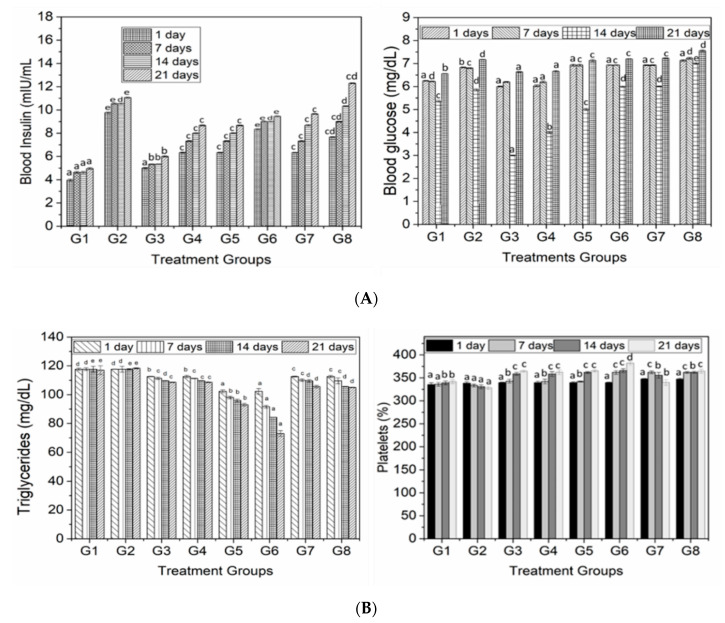
(**A**) Blood serum analysis of rats fed with different concentrations of polyphenol extract from guava leaves, seeds and pulp. Lowercase letters (a–e) show the significant difference (*p* < 0.05) among treatments within the same time. (**B**) Triglycerides and platelets of mice fed with different concentrations of polyphenol extract from guava leaves, seeds and pulp. Lowercase letters (a–e) show the significant difference (*p* < 0.05) among treatments within the same time.

The results regarding Triglycerides are shown in Figure 2B. The highest mean value (117.6 mg/dL) of triglyceride of rat groups was found in group 2. The lowest triglyceride value (87.5 mg/dL) was observed in group 6. The results for TC (Table 2) exhibited a significant change in the plasma TC level of experimental rats among different diet groups during the entire study interval. TC content was found to be higher in a control group while decreased in groups supplemented with extracts of different concentrations. A significant difference was noticed among the treatment groups within the same time of supplementation. It was also noticed that this difference was more significant (*p* < 0.05) when rats were fed with high concentration of guava pulp and for three weeks. Overall, this difference was more prominent in G4 fed with pulp extract. On the other hand, the difference between G5, G6 and G7 was not significant with respect to the control group. 

The results for HDL are presented in Table 2. The increasing trend was observed for groups fed with extracts while control groups showed decreased HDL values. On the other hand, LDL content was decreased the same as TC for treated groups, while it was increased in control groups. Regarding the number of days, no significant difference was observed at the start of experiment; however, the increased trend was observed as number of weeks increased and it was highest at 3 weeks of supplementation. LDL values were decreased with the supplementation of phenolic extracts, while, in the controls, especially the –ive control, it was not changed throughout the experimentation. However, with the supplementation of guava polyphenolic extract for 3 weeks, especially that from guava pulp and leaves, LDL levels significantly decreased. 

The results for blood glucose level in rats with and without the supplementation of polyphenolic extracts are presented in Figure 2A. The findings reveal that control groups having diabetes mice had high level of blood glucose indicating the diabetic symptoms. On the other hand, with the addition of guava extracts in treatment groups, the glucose level significantly decreased (*p* < 0.05) with the supplementation of extract for a prescribed period of time, and this difference was more obvious when rats were fed with guava pulp extract followed by leaves and seed extract. In some treatments, e.g., G6, the glucose level was higher, but it was not significantly higher than the control groups. However, in all groups that were fed with guava extract showed significantly decreased in glucose level but control groups remained unchanged with respect to glucose level even after 4 week of diet. 

Regarding the results of blood insulin (Figure 2A), a similar trend was observed for blood glucose; however, blood insulin was much lower compared to the +ive control. Interesting results were revealed when G5, G6 and G7 did not show any significant difference due to the supplementation of guava leaves and seed extract. Although insulin was decreased, it was not less than the negative control; however, it was just near to negative control but considerably less than the positive control, indicating the antidiabetic potential of polyphenols. 

### 3.5. Hematological Studies

The results for PCV are presented in Table 3. The results show that PCV increased in groups treated with guava extract and this was significantly higher in groups that were fed with guava pulp (G4). The supplementation of guava seed and leaf extract also increased the PCV and was especially significant at 21 days, but this was less than guava pulp extract. A similar trend was observed for MCV, and it was revealed that MCV was higher in groups supplemented with guava extract. This significant difference was more obvious when guava leaves (G6) were fed for 21 days. Meanwhile, the groups fed with guava seeds showed a lesser increase than the pulp, but still these were more than the control groups. Interestingly, there was a statistically significant difference between the +ive and –ive control groups even at start of experimentation, indicating the initial difference between both control groups.

**Figure 3:**
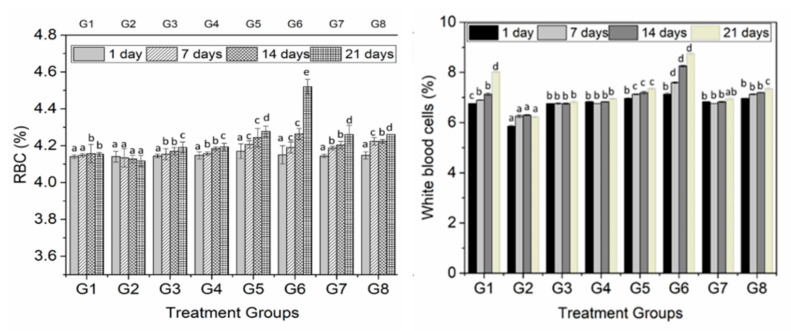
Blood serum analysis of rats fed with different concentration of polyphenol extract of guava leaves, seeds, and pulp. Lowercase letters (a–c) show the significant difference (*p* < 0.05) among treatments within the same time.

The results for hemoglobin level in rats fed with different guava extracts were more significant at 21 days of supplementation. On the other hand, in the control group (G2), the level of hemoglobin was not significantly increased. The results suggest the significant influence of extract on diabetes levels that was significantly decreased in terms of blood glucose and insulin levels. The results for platelets, RBC and WBC with and without the addition of guava extract are shown in Figure 2B and Figure 3, respectively. The findings exhibit a significant (*p* < 0.05) change in the WBC contents of experimental rats among different diet groups during the entire study interval. The results were also found to be significant at 0 and 21 days of feeding interval. The minimum RBC level (4.12%) was found in Group 2, while the maximum RBC (4.22%) concentration was observed in the rats of group 5. The level of RBCs increased from day 0 to day 21 (4.14 to 4.24%). 

The incorporation of diets with different plant parts of guava exhibited a significant influence on the platelets of diabetic rats at day 21’s study interval. The highest mean value (358.92%) of platelets of rat groups was found in Group 8 (Figure 2B). The lowest platelets value (332.67%) shown by group 2 was from the +ive control. On the other hand, these parameters (WBC and RBC) were decreased in diabetic mice while increased in negative control group, but this increase was not very significant compared to other treatment groups.

## 4. Discussion

The screening of plants and their different parts for health-promoting activities has been a prime priority due to their natural quality and the fact that they produce the least side effects after supplementation. Previous studies revealed that guava (133 genera and about 4000 species) has been identified and used as a functional food, while it has been known as the poor man’s apple of the tropics [10]. The different parts (leaves, pulp, bark and seeds) of guava have been proved to be antibacterial [4], against infectious disease, antiproliferative [19], antispasmodic, and immunostimulant [20] due to them having a broad spectrum of active ingredients such as polysaccharides, vitamins, minerals, polyphenols, antioxidants, carotenoids, lutein, zeaxanthine, tannins, saponins, essential oils, pectin, dietary fiber and fatty acids [10]. All these compositional attributes make guava suitable for pharmaceutical applications. 

In the present investigation, the antidiabetic potential of polyphenolic extract of different parts (pulp, seeds and leaves) was assessed using diabetic mice. Prior to this assessment, the proximate composition and polyphenolic and antioxidant activities of the above-mentioned parts were evaluated (Table 1), and they were found to be highest in leaves followed by pulp and seeds. These results were agreeing with the previous study [4] conducted on guava leaves, which also approved the suitability of methanol extraction method. Regarding composition, the results of vitamin C (Table 1) were highest in pulp powder, and this amount was even more than any other parts [21]; however, it was less than unripe fruits mentioned in the same study. Vitamin C composition in pulp was higher than the content of orange juice [22], indicating the richest source of vitamin C. 

After assessing the proximate composition and total phenolic contents, the phenolic extract of pulp, seeds and leaves was supplemented to diabetic mice along with control diet and control group. Parameters such as body weight, feed intake, blood glucose and insulin, triglycerides, PCV, RCB, WBC and platelets were measured at 1, 7, 14 and 21 days. The concentrations (200 and 250 mg/kg.bw) of the extract of pulp, seeds and leaves were fed along with natural diet. Weight gain as a measure of the nutritive value of dietary proteins has been most popular. It estimates the retention of nitrogen in the body for maintenance and growth. The overall growth of the body measured as a gain in weight or length or both was often used as an estimation of a gain in body nitrogen. The correlation between a gain in weight and a gain in nitrogen was good in the rats. Since the feed intake was increased in supplemented groups, body weight was also increased; however, this difference was more prominent based on the number of days compared with the treatment groups (Figure 1). 

The antidiabetic potential against various extracts of polyphenols was assessed in diabetic mice. The results were analyzed using one-way ANOVA and Duncan Multiple range’s test at a significant level of *p* < 0.05. Moreover, the significant difference between treatment groups was determined within the same time using mean values of experimentation. Regarding the results of TC, HDL and LDL, it was revealed that addition of polyphenol extract improved the blood lipid profile, which is an important indicator of diabetes. Previous studies have suggested that decreased TC and LDL while increased HDL resulted in cardiovascular diseases and atherosclerosis [23]. Elevated serum HDL-C levels decreased atherosclerosis, as HDL-C in serum is believed to contribute to the translocation of excessive cholesterol, further catabolic cholesterol, from peripheral tissue to the liver [24]. Furthermore, among the studied extracts, the polyphenols from pulp showed a higher positive response, followed by leaves, indicating the strong hypoglycemic effect. Our results are well connected with a previous report [25], which also reported the polyphenol extract fed to the mice resulted in the decreased TC and LDL. This might be due the fact that the reduction of TC induced by polyphenols might decrease the cholesterol absorption and biosynthesis while increasing fecal bile acid as well as cholesterol excretion [26]. 

The triglycerides, blood sugar and insulin are considered as remarkable parameters while studying diabetes [27]. These all have a strong influence in the elevation or decrease of diabetes with the most preferential is blood sugar. High blood sugar causes hyperglycemia in diabetic patients; thus, the control released of glucose is very necessary in order to control the diabetes [21]. Regarding blood glucose level, as indicated in Figure 2A,B, blood glucose level was significantly (*p* < 0.05) reduced, especially at 14 days with the treatments groups fed with pulp extract followed by leaves. Same as blood insulin, blood glucose level was decreased with the supplementation of extract compared to the positive control. This decrease in blood glucose level might be due to the inhibition of α-amylase and α-glucosidase activities induced by polyphenols and fiber, as indicated by a previous report [28]. This inhibition has a long-term relation in the management of diabetes as compared to comparable with the drug acarbose, which upon high concentration caused pancreatic exhaustion, insulin resistance, glucose intolerance and an increased insulin demand [29]. A considerable decrease in blood insulin was observed, especially with a low dose of polyphenol extract from pulp. Previous studies have also reported the inhibition of α-amylase by means of polyphenols such as from apples [18,30], berries [31] and green tea [28]. Another possibility for the reduction of blood glucose is the inhibitory action of polyphenols that reduced the glucose transport reported by many studies [28,30,32]. 

Blood is a bodily fluid that delivers necessary substances, such as oxygen and nutrients (glucose, amino acids and fatty acids), to the tissues and cells. It also transports metabolic waste products to the kidneys and liver to be filtered and excreted. The RBC also called erythrocytes is one type of blood cell that delivers oxygen (O_2_) to the body tissues and cells. Low RBCs is associated with anemia and sometimes toxicity. Blood constituents (hemoglobin, RBC, WHC and platelets) have a significant role in order to maintain diabetes and a major role in the transportation of respiratory gases and nutrients [33]. Regarding haematological parameters, it was found that the supplementation of polyphenol extract has a positive influence on the hemoglobin, RBC and WBC of diabetic mice. On the other hand, these parameters were decreased in diabetic mice while increased in the negative control group, but this increase was not very significant compared to other treatment groups. The increase in the blood haematological parameters of diabetic mice is an indication that guava extracts especially from pulp and leaves have the potential to provoke erythropoietic release from a kidney, which behaves as a humoral regulator for the production of RBC [34]. These results indulged that polyphenols have an ability to raise the oxygen carrying capability of blood. 

The supplementation of phenolic extracts to diabetic mice increased the RBC and WBC, indicating the improved immune system. Previous studies also reported the improved immune system through the supplementation of polyphenols, such as from grapes [35], olive waste [36], and green tea [11]. The results for MCV, PCV and hemoglobin were significant only when 250 mg/kg.bw of polyphenols form guava pulp was supplemented for 21 days while in the +ive control, these parameters were decreased compared to all other treatment groups. The results are convincing that the extract of polyphenol has a tremendous effect on levels of PCV, MCV and hemoglobin and the values were just close to control groups. However, these values were much higher than the +ive control, highlighting the influential consequences of polyphenol extracts. The increase in PCV, MCV and hemoglobin levels has a great role in anemia diagnosis in animals.

## 5. Conclusions

The present study investigated the antidiabetic potential of polyphenol from a guava’s different parts (pulp, seeds and leaves). The quantification and screening of polyphenols from guava for the purpose of diabetes is an encouraging step for diabetes management. The results, such as, decreased TC and LDL and increased HDL, are convincing pieces of evidence, which have potential in order to apply for pharmaceutical applications. Moreover, the controlled blood glucose release and decreased insulin are the indication of fruitful supplementation. Furthermore, PCV, MCV and hemoglobin levels were improved, highlighting the strong antidiabetic effect of polyphenol. Summarizing this study, among the studied extracts, polyphenols from pulp were found to be more effective against diabetes followed by leaves. Thus, this study suggests the supplementation of polyphenol extract from guava pulp with a dose of 250 mg/kg.bw as an alternative to diabetes drugs. Beside this, future work is needed in order to check the antidiabetic effect of these extracts at a fasting stage and diurnal dose supplementation. This will not only help to design an effective therapeutic but will also help to design a diurnal dependent dose with an improved synergistic effect.

## Figures and Tables

**Table 1:** Proximate composition and total phenolic content of guava pulp, leaves and seeds.

Samples/Parameters	Leaves	Seeds	Pulp
Moisture (%)	82.47 ± 2.10 ^b^	46.22 ± 1.50 ^a^	52.18± 1.60 ^ab^
Ash (%)	3.64 ± 0.05 ^c^	3.15 ± 0.10 ^b^	2.42 ± 0.20 ^a^
Fat (%)	0.62 ± 0.23 ^a^	7.94 ± 1.23 ^b^	6.54 ± 1.11 ^b^
Protein (%)	18.53 ± 2.29 ^b^	13.31 ± 1.34 ^a^	10.64 ± 1.66 ^a^
Carbohydrates (%)	12.74 ± 1.87 ^c^	3.06 ± 1.47 ^a^	8.57 ± 1.52 ^b^
Vit. C (mg)	103.05 ± 4.59 ^b^	87.43 ± 5.72 ^a^	116.17 ± 6.32 ^c^
Total phenolic compounds (mgGAE/g)	1717 ± 6.43 ^b^	344 ± 3.77 ^a^	383 ± 9.32 ^a^
Antioxidant activity (%)	234 ± 7.57 ^b^	89 ± 6.11 ^a^	365 ± 8.65 ^c^

^abc^ Superscript letters are found to be significantly different using one-way ANOVA and the Duncan multiple range test at a significant level of *p* < 0.05.

**Table 2:** Biochemical and blood serum characteristics of mice fed with and without the supplementation of guava extracts during 3 weeks of administration.

Groups	Total Cholesterol (mg/dL)				High Density Lipoprotein (HDL) (mg/dL)				Low Density Lipoproteins (LDL) (mg/dL)			
	0	7	14	21	0	7	14	21	0	7	14	21
G_1_	57.1 ^a^	57.8 ^a^	57.8 ^a^	57.9 ^a^	22.6 ^a^	22.6 ^a^	22.3 ^b^	22.6 ^b^	23.4 ^a^	23.5 ^d^	23.4 ^d^	23.4 ^d^
G_2_	57.8 ^b^	57.8 ^a^	56.9 ^b^	54.3 ^b^	22.0 ^b^	20.6 ^a^	19.6 ^a^	19.3 ^a^	23.5 ^a^	23.5 ^d^	23.4 ^d^	23.6 ^d^
G_3_	57.6 ^b^	54.7 ^b^	54.2 ^b^	53.8 ^c^	22.0 ^b^	24.3 ^b^	26.0 ^c^	28.3 ^d^	22.5 ^a^	22.6 ^c^	21.9 ^c^	21.7 ^b^
G_4_	57.6 ^b^	54.0 ^b^	54.8 ^b^	52.3 ^d^	22.0 ^b^	25.3 ^b^	26.3 ^c^	30.3 ^e^	22.5 ^a^	22.6 ^c^	21.9 ^c^	17.0 ^a^
G_5_	57.2 ^b^	54.6 ^b^	53.4 ^b^	53.8 ^c^	22.0 ^b^	25.6 ^b^	27.0 ^d^	27.3 ^d^	23.4 ^a^	19.6 ^b^	19.2 ^b^	21.6 ^b^
G_6_	57.4 ^b^	53.9 ^c^	53.9 ^c^	53.7 ^c^	22.3 ^b^	25.6 ^c^	27.6 ^d^	30.3 ^e^	23.2 ^a^	19.3 ^a^	16.8 ^a^	22.6 ^c^
G_7_	57.6 ^b^	54.7 ^b^	54.9 ^b^	53.8 ^c^	22.0^b^	24.6 ^b^	25.3 ^c^	25.3 ^c^	23.4 ^a^	22.0 ^c^	21.8 ^c^	21.1 ^b^
G_8_	57.6 ^b^	53.4 ^c^	52.8 ^c^	54.9 ^b^	22.3 ^b^	25.3 ^c^	26.3 ^c^	28.3 ^d^	23.2 ^a^	21.8 ^c^	21.1 ^c^	21.0 ^b^

Reported values are mean values from triplicates analysis. ^abc^ Lowercase letters (a–e) in columns show the significant difference (*p* < 0.05) among treatments within the same time.

**Table 3:** Packed cell volume (PCV), mean corpuscular volume (MCV) and the hemoglobin levels of diabetic and control mice fed with different concentrations of polyphenol extract of guava leaves, pulp and seeds.

Groups	PCV (%)				MCV (g/kg)				Hemoglobin Level (%)			
	0	7	14	21	0	7	14	21	0	7	14	21
G_1_	38.6 ^a^	39.3 ^a^	40.3 ^a^	41.0 ^a^	92.6 ^a^	92.6 ^a^	94.3 ^a^	94.6 ^a^	10.0 ^b^	10.6 ^b^	11.3 ^b^	12.0 ^b^
G_2_	39.3 ^b^	39.6 ^a^	40.6 ^a^	41.6 ^b^	94.0 ^b^	92.6 ^b^	94.3 ^a^	98.0 ^b^	9.0 ^a^	10.0 ^a^	10.0 ^a^	10.6 ^a^
G_3_	39.3 ^b^	40.3 ^b^	41.0 ^b^	44.6^c^	94.0 ^b^	97.0 ^c^	98.0 ^c^	99.0 ^c^	9.6 ^a^	11.6 ^b^	11.6 ^c^	12.3 ^c^
G_4_	39.3 ^b^	40.6 ^b^	41.3 ^b^	46.0^d^	94.0 ^b^	97.6 ^c^	99.3 ^cd^	106.0 ^f^	9.3 ^a^	11.8 ^c^	11.9 ^e^	12.9 ^f^
G_5_	39.4 ^b^	42.6 ^d^	44.3 ^c^	42.0^a^	94.0 ^b^	97.6 ^c^	103.3 ^d^	108.6 ^d^	9.3 ^a^	11.8 ^c^	11.8 ^d^	12.6 ^d^
G_6_	39.3 ^b^	41.6 ^c^	44.3 ^c^	45.0^c^	94.6 ^b^	102.3 ^e^	106.6 ^e^	109.0 ^g^	9.6 ^a^	11.9 ^d^	11.9 ^e^	12.7 ^de^
G_7_	39.2 ^b^	40.3 ^b^	41.0 ^b^	41.6 ^b^	94.6 ^b^	97.6 ^c^	99.3 ^cd^	101.0 ^d^	9.6 ^a^	11.6 ^b^	11.9 ^e^	12.3 ^c^
G_8_	39.5 ^b^	40.6 ^b^	41.3 ^b^	42.0 ^a^	94.3 ^b^	98.0 ^d^	100.6 ^d^	102.6 ^e^	9.3 ^a^	11.1 ^cd^	11.9 ^e^	12.8 ^e^

^abc^ Lowercase letters (a–g) in columns show the significant difference (*p* < 0.05) among treatments within the same time.
